# IgA Vasculitis Associated With Levofloxacin Use in an Adult Female: A Case Report

**DOI:** 10.7759/cureus.71567

**Published:** 2024-10-15

**Authors:** Karen A Gómez Contreras, María E Alonzo Canul, Ana L Mena Rodríguez, Melissa G Castillo Baas, David A Vargas Gutiérrez

**Affiliations:** 1 Internal Medicine, Clínica Hospital Mérida, Mérida, MEX; 2 Dermatology, Clínica Hospital Mérida, Mérida, MEX; 3 Pathological Anatomy, Clínica Hospital Mérida, Mérida, MEX; 4 Rheumatology, Hospital General de México, Mexico City, MEX

**Keywords:** adult vasculitis, drug-induced cutaneous vasculitis, fluoroquinolones, iga vasculitis, infections, levofloxacin, upper respiratory tract infections

## Abstract

Immunoglobulin A (IgA) vasculitis, once known as Henoch-Schönlein purpura in recognition of the physicians who first identified it, is an acute form of vasculitis, associated with a deposition of immune complexes, self-limited that affects small-caliber vessels. It usually occurs in children and rarely in adults. This disease can be induced by multiple factors such as exposure to certain infectious antigens such as viruses or bacteria, drugs, and toxins, and even genetic predisposition has been described. Although the use of some antibiotics is known as a risk factor for the development of IgA vasculitis, it may not be considered when approaching and treating an adult with a related clinical condition, since its frequency is rare. We present the case of a 26-year-old woman with a history of an upper respiratory tract infection who required treatment with levofloxacin and subsequently developed palpable purpura.

## Introduction

Immunoglobulin A (IgA) vasculitis is an acute systemic vasculitis caused by perivascular deposits of IgA immune complexes in small vessels and the activation of neutrophils where both genetic predisposition and exposure to certain infectious antigens, such as viruses or bacteria and drugs or toxins, can trigger the disease. Clinically, it presents with a palpable and painless purpuric rash that is usually the first sign of most patients, with a usual distribution in a symmetrical pattern involving the extensor surfaces of the lower legs, buttocks, and arms. Later, it can extend to the extensor areas of the forearms, cheeks, and ears. In most cases, the face, palms of the hands, soles of the feet, and mucous membranes are spared. It can also occur limited to the skin or associated with arthralgias that predominate in large joints of the lower extremities or abdominal pain, which may occur two weeks before the eruption. New lesions may develop over a period of eight weeks. Meanwhile, established lesions change their skin tone from red to purple and disappear in approximately 10 days [1-3]. Up to 55% of patients may have kidney disease manifesting with hematuria or proteinuria, and on rare occasions, the central nervous system and lungs may also be affected [1,4].

This disease is more common in childhood, with an annual incidence of 18-30 cases per 100,000 children, and becomes rare after the age of 15, with only 10% of cases, which is why it is rarely reported in adults. Timely diagnosis by first-contact doctors is important to treat and to monitor possible serious complications such as renal failure, intussusception, gastrointestinal bleeding, intracranial hemorrhage, pleural effusion, pulmonary hemorrhage, and testicular torsion [4].

This case report informs us about the existence of a pathology mainly described in children, which affects adult patients less frequently, for which no specific cause has been documented, but rather a group of probable causes, which generates an impact on the health of patients and could become preventable [5]. We present the case of a 26-year-old female patient with cutaneous IgA vasculitis attributed to quinolone exposure. It is likely that this patient has a genetic susceptibility to developing immune-mediated vasculitis and that the drug exposure acted as a second hit. We cannot rule out the possibility that the patient's chronic renal damage was caused by an unidentified IgA vasculitis and that the drug exposure only caused a flare vasculitis, as suggested by some authors [6].

## Case presentation

The patient is a 26-year-old Mexican woman, with a four-year history of arterial hypertension treated with telmisartan, amlodipine, and metoprolol. Moreover, she has chronic kidney disease of unknown etiology in stage 5 of the Kidney Disease Improving Global Outcomes (KDIGO) diagnosed also four years ago. A renal biopsy was performed in which diffuse glomerulosclerosis was identified. The patient is currently on kidney replacement therapy with automated peritoneal dialysis and medical treatment with furosemide, iron, folic acid, and erythropoietin for chronic anemia. Her condition began 15 days prior with the presence of non-quantified fever and nasal congestion. She was treated with levofloxacin 500 mg daily for five days by her primary care physician, with partial improvement in her respiratory symptoms.

The patient sought further medical evaluation, as she developed odynophagia, asthenia, and mild crampy abdominal pain. It was accompanied by dermatoses of two days of evolution, which began in an ascending presentation from the feet to the lower third of the thighs in both legs, referred to as erythematous macules, causing slight burning in the entire affected surface. She denied ever having these lesions before.

**Figure 1:**
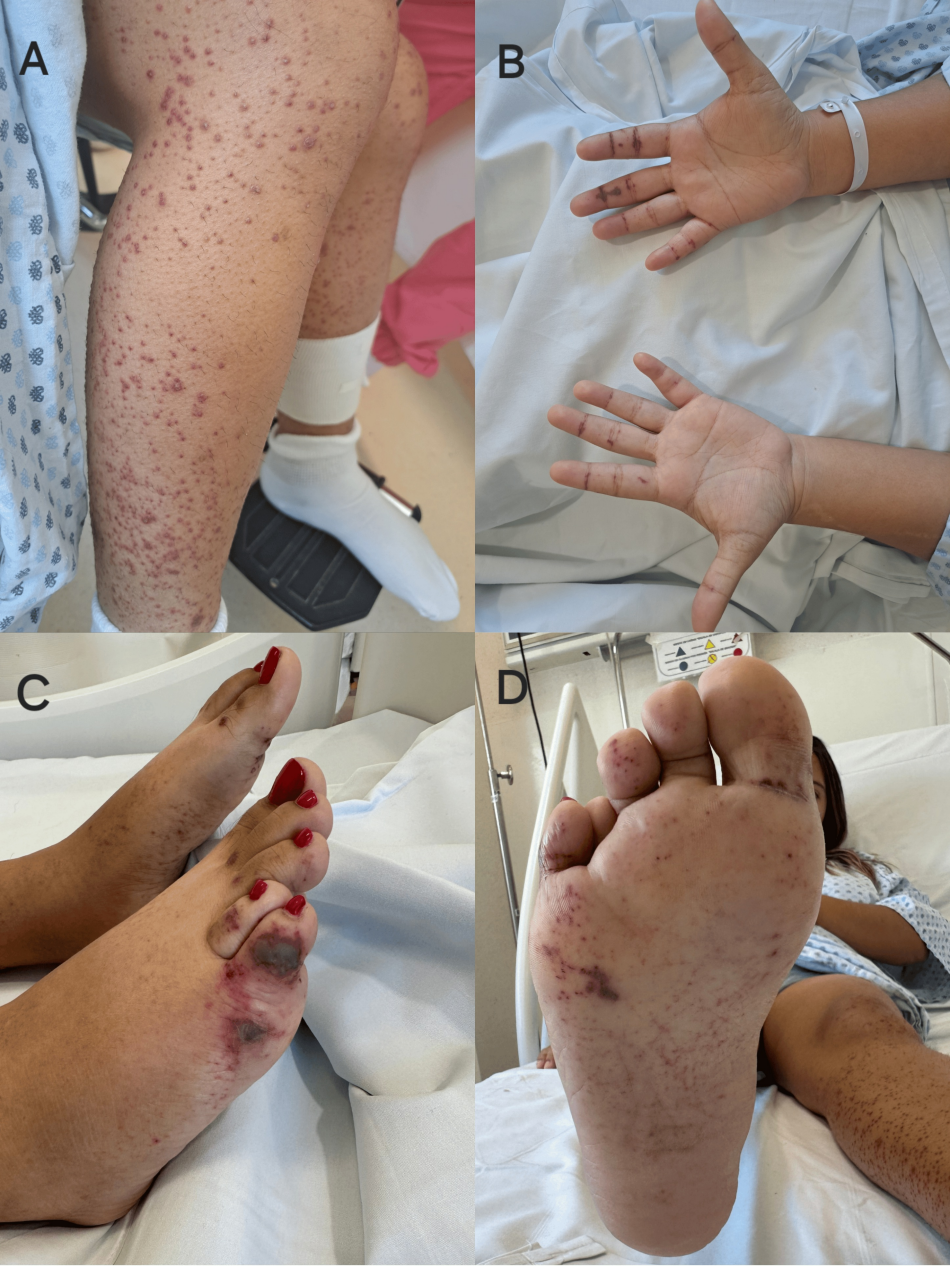
Dermatological examination 24 hours after admission. Skin lesions: multiple raised, well-circumscribed purpuric lesions on the leg (A). Presence of hemorrhagic-necrotic lesions on palms (B) and in the lateral area of ​​the fifth toe of the right foot (C) and presence of hemorrhagic-necrotic lesions and punctate purpuric lesions in soles of the feet (D).

She was admitted for further tests. On evaluation, we find a disseminated rash on the lower extremities, with multiple raised, well-circumscribed palpable vasculitic lesions of variable sizes up to 15 mm of red to violet hue. After 24 hours, the lesions spread to the trunk, upper extremities, palms, and sole of the foot (Figure 1). She developed arthralgias located in the ankles, knees, shoulders, and phalanges of the hands.

The blood test showed a chronic hypochromic normocytic anemia secondary to iron deficiency in the previous treatment, which did not worsen during the development of vasculitis, moderate hyponatremia, and increase of urea and creatinine with an estimated glomerular filtration rate of 4 mL/min/1.73 m^2^, mild thrombocytosis, and no biochemical signs of coagulopathy. There was no evidence of hemorrhage at the clinic evaluation. Vasculitis was suspected due to the history of having had an upper respiratory tract infection associated with palpable purpura and the topography of the lesions. Skin biopsy, immunofluorescence, and the test in Table 1 were requested to perform differential diagnoses with autoimmune diseases, vasculitis due to the immune complexes associated with drugs such as leukocytoclastic vasculitis, and post-infectious thrombocytopenic purpura.

**Table 1:** Laboratory tests obtained on the first day of hospitalization. anti-dsDNA: anti-double-stranded DNA; ELISA: enzyme-linked immunosorbent assay; HIV: human immunodeficiency virus

Laboratory test	Patient's findings	Reference range
Anti-dsDNA	8.63 UI/mL	Negative <20 UI/mL
Antinuclear antibodies (ELISA)	0.08 UI/mL	Negative <1.50 UI/mL
Myeloperoxidase anti-neutrophil cytoplasmic antibody	1.69 UR/mL	Negative <20 UR/mL
Proteinase 3-specific anti-neutrophil cytoplasmic antibody-associated vasculitis	2.64 UR/mL	Negative <20 UR/mL
C3 complement	1.700 g/L	0.830-1.930 g/L
C4 complement	0.280 g/L	0.150-0.570 g/L
Rheumatoid factor	8 UI/mL	≤20 UI/mL
C-reactive protein	48 mg/dL	Positive ≥48 mg/dL
Erythrocyte sedimentation rate	39 mm/hr	1.00-15.00 mm/hr
ELISA test for HIV	Negative	
Hepatitis B surface antigen	Non-reactive	
Hepatitis C virus antibody test	Non-reactive	
Epstein-Barr virus by polymerase chain reaction	Non-reactive	
Polymerase chain reaction to detect cytomegalovirus	Undetected	
Anti-Epstein-Barr IgG antibodies	568.00 S/CO	Negative <20; positive ≥20
Anti-Epstein-Barr IgM antibodies	<10.000 U/mL	Negative <20 U/mL; gray zone 20-40 U/mL; positive >20 U/mL
Throat swab culture	Development of normal flora	

**Figure 2:**
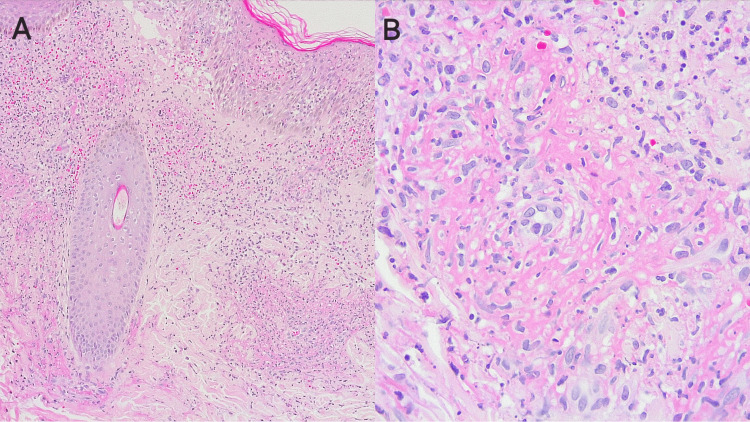
Skin biopsy of a vasculitic lesion on the external lateral aspect of the distal third of the left leg. 4× magnification of panoramic skin image: an increase in inflammatory infiltrate, mainly in the dermis (A). 40× magnification: an arteriolar vessel with perivascular inflammation composed of neutrophils, cellular debris, fibrin, and extravasation of erythrocytes (B).

**Figure 3:**
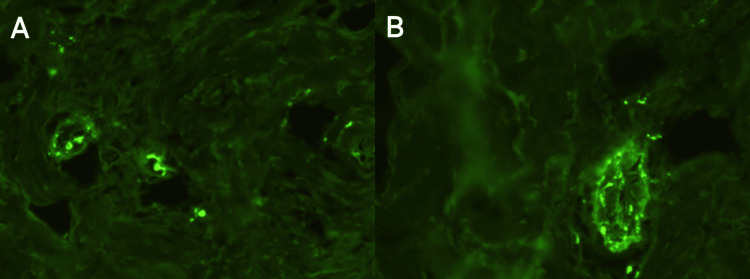
Direct skin immunofluorescence. In the 4× magnification panoramic image of the immunofluorescence histological assay, IgA deposits are identified in most dermal capillaries (A). At 40× magnification, there is the presence of IgA deposits in the vascular wall (B).

An excisional biopsy was performed on two purpuric lesions less than 24 hours after their appearance. They were taken from the left leg and the third finger of the left hand. The report showed a superficial and deep necrotizing leukocytoclastic vasculitis (Figure 2). Immunofluorescence was positive for IgA (Figure 3).

Once the biopsy was performed, systemic glucocorticoid was started at intermediate doses equivalent to prednisone. In the next 24 hours, the patient reported improvement in joint pain, less edema in both hands, and no more new purpuric lesions in the skin. Once the patient was in no pain, there were no new purpuric lesions, and the first lesions that appeared improved, we decided to discharge the patient with oral prednisone. In the two-week follow-up, complete resolution of the lesions is evident.

## Discussion

IgA vasculitis, formerly known as Henoch-Schönlein purpura, was first described in 1802 by William Heberden, and in 1837, Johann Lukas Schönlein associated purpura with arthralgia and arthritis. In 1874, Eduard Henoch reported it in children along with symptoms such as abdominal pain, bloody diarrhea, and joint pain [7]. IgA vasculitis has a worldwide distribution, where 90% of cases occur in children between three and 15 years, with an annual incidence of 18-30 cases per 100,000 children, and is rare in adults with an annual incidence of 0.1-1.8 per 100,000 people [5,8].

IgA vasculitis is an acute disorder caused by perivascular deposits of IgA immune complexes and the recruitment of neutrophils that in turn cause fibrinoid destruction and leukocytoclastic vasculitis of small vessels of the skin, kidneys, gastrointestinal tract, and joints and occasionally can affect the lungs and central nervous system. Multiple causes associated with IgA vasculitis have been identified, the most common being a history of upper respiratory tract infection where exposure to viruses, bacteria, drugs, or toxins can trigger the disease. Drugs related to IgA vasculitis include quinolones, angiotensin-converting enzyme inhibitors, angiotensin II receptor antagonists (such as losartan), clarithromycin, and some non-steroidal anti-inflammatory drugs [9,10]. Also, an association with infectious mononucleosis, parainfluenza virus, influenza virus, respiratory syncytial virus (RSV), adenovirus, COVID-19 infections, group A *Streptococcus* (the most common), *Haemophilus parainfluenzae*, and methicillin-resistant *Staphylococcus aureus* (MRSA) was reported [2,4]. Even genetic predisposition has been described with the HLA-DQA1 and DQB1 intergenic zone, the HLA-DRB1*01:11/B1*13 loci, and the DQA1*01:01/DQB1*05:01/DRB1*01:01 haplotype [11].

Although the triggers for this condition are recognized, the underlying cause of IgA vasculitis remains unknown. As it is an uncommon illness in adults, fewer triggering factors have been associated in addition to different clinical manifestations. Pillebout questioned the association between IgA vasculitis and IgA nephropathy and analyzed a wide variety of presentations, where some authors have agreed on the possibility that both belong to the same entity and that they can occur synchronously or out of time. In our patient, we suspect that the cutaneous IgA vasculitis is related to an IgA vasculitis previously confined to the kidney, which was not previously detected and when subsequently exposed to the drug triggered an episode of systemic activity [6].

In this case, it is striking that, unlike children, adult patients usually present purpuric lesions associated with blisters or hemorrhagic-necrotic lesions that have been described in up to 60% of adult patients. There have been described cases in adults with potentially life-threatening manifestations that have required intensive immunosuppression [12,13]. In our case, the patient presented multiple hemorrhagic-necrotic lesions that predominated in the palms of the hands and soles of the feet. As the purpuric lesions in the skin were presented, the initial differential diagnosis included Henoch-Schönlein purpura or IgA vasculitis, vasculitis due to immune complexes associated with drugs such as leukocytoclastic vasculitis, or post-infectious thrombocytopenic purpura as diagnostic possibilities. We considered the possibility of IgA vasculitis as most possible because she presented three elements of the classical tetrad: palpable purpura without thrombocytopenia or coagulopathy, arthralgia, and abdominal pain. In this case, we could not measure proteinuria due to oliguria associated with low glomerular reserve. A skin biopsy and immunofluorescence were performed to confirm the diagnosis due to the low incidence of IgA vasculitis in adults.

It is known that a history associated with IgA vasculitis is respiratory tract infections and that levofloxacin can generate hypersensitivity reactions. Cases of vasculitis associated with fluoroquinolones have also been reported, so according to the probability scale of adverse drug reactions from Naranjo et al., we obtained a score of 2, determining that levofloxacin was a possible cause of vasculitis [14,15]. In other reports, ciprofloxacin has been the predominant fluoroquinolone associated with cutaneous vasculitis, and less frequent were ofloxacin and levofloxacin. However, similarities between these fluoroquinolones have been highlighted, for example, levofloxacin is the L-enantiomer of ofloxacin. The similar adverse effects between levofloxacin and ciprofloxacin, such as cutaneous vasculitis, have been explained by their structural characteristics, since in the seventh position of ciprofloxacin, there is an unsubstituted piperazine ring and in the seventh position of levofloxacin, there is 4-methylpiperazine, unlike the great variability that other fluoroquinolones present in their structure in the seventh position of the molecule [16]. The mechanism responsible for drug-associated immune complex vasculitis is unclear; however, from the immunofluorescence results, we suggest that the drug is likely to stimulate an immune response by acting as a hapten or that may have even exacerbated a systemic manifestation of IgA nephropathy.

Levofloxacin has been considered a useful drug for the treatment of respiratory tract infections; however, it has been warned that the use of fluoroquinolones, in doses higher than those recommended, is associated with a significant increase in side effects and hypersensitivity in patients with severe renal failure, that is, patients with an estimated glomerular filtration rate of <30 mL/min/1.73 m^2^ or those receiving dialysis [17]. In the case of our patient, who is being treated with a dialysis modality, as long as the indication for levofloxacin has been justified, the recommended dose is 500 mg every 48 hours to reach its minimum inhibitory concentration and reduce the risk of complications, since even in healthy patients levofloxacin at a dose of 500 mg every 24 hours has been associated with vasculitis [18].

As in previous case reports, our patient made a complete recovery with prednisone treatment and levofloxacin discontinuation [16,19]. We cannot be completely sure that levofloxacin was the direct cause of the development of vasculitis in the patient since she also has an upper respiratory tract infection as a risk factor for developing this condition. However, we consider it of great importance to highlight the risks that determine the administration of antibiotics in primary care without considering the comorbidities of the patients, since in the case of our patient, the purpuric manifestations were triggered by the administration of the antimicrobial. We also want to highlight the relevance of considering IgA vasculitis as a differential diagnosis in adult patients with this type of clinical symptoms, given that timely diagnosis and treatment are crucial to reducing serious complications reported in adult patients such as purpura fulminans and lung and kidney damage [13,18,19].

## Conclusions

IgA vasculitis is an uncommon condition; therefore, the clinical characteristics to make the diagnosis must be clear, such as palpable purpura without thrombocytopenia or coagulopathy, arthralgias, abdominal pain, and nephropathy. This is important because early diagnosis and treatment are vital to avoid renal involvement. This case highlights the need for primary care physicians to be alert to this diagnostic possibility to intervene effectively and early, as well as to raise awareness about fluoroquinolone dosing recommendations in patients with chronic kidney disease since higher concentrations of this drug may increase the incidence of adverse effects. If it is determined that the benefits of fluoroquinolone use outweigh the potential risks, it is crucial to initiate treatment at a reduced dose appropriate for the patient's renal function. Patients should be educated about possible signs of toxicity and monitored closely at the start of treatment to detect any early adverse effects.
